# 

**Published:** 1898-04

**Authors:** 


### CONTENTS:

### SELECTIONS.

Article I.—Antiseptics and Germicides in Dentistry. By G. S. Martin, D. D. S., L. D. S. .... 529

" II.—Four Classes of Dentists, .... 534

" III.—The Natural Law of Cure. By A. P. Betts, M. D. .... 539

" IV.—Habits and Products of Germs. By Ernest B. Sangree, A. M., M. D. .... 544

" V.—To Remove Enamel Previous to Crowning. By H. H. Johnson, D. D. S. .... 547

" VI.—The Education Features of the Dental Association. By J. Percy Corley .... 549

" VII.—Oral Hygiene. By Eugene S. Talbot, M. D., D. D. S. 551

" VIII.—The Use of Soft Foil. By C. C. Chittenden, D. D. S. 557

### EDITORIAL, ETC.

Proposed Dental Law .... 562

Questions Given to Applicants for Certificates to Practice Dentistry in Maryland .... 568

### COMMUNICATED

American Medical Association .... 571

### MONTHLY SUMMARY.

Hints .... 572

Prescription Writing .... 573

Discolored Teeth .... 574

Shall the Dentist Advertise? .... 575

Students Practicing Dentistry .... 576

Fastidious Operating .... 576

